# Weight Change and All-Cause Mortality in Later Life: Findings From the National Survey of the Japanese Elderly

**DOI:** 10.1093/geroni/igaa057.1212

**Published:** 2020-12-16

**Authors:** Hiroshi Murayama, Jersey Liang, Benjamin Shaw, Anda Botoseneanu, Erika Kobayashi, Taro Fukaya, Shoji Shinkai

**Affiliations:** 1 Tokyo Metropolitan Institute of Gerontology, Tokyo, Japan; 2 University of Michigan, Ann Arbor, Michigan, United States; 3 State University of New York, Rensselaer, New York, United States; 4 University of Michigan, Dearborn, Michigan, United States

## Abstract

Recent studies predominantly in Western populations suggest that both weight gain and weight loss are associated with increased mortality risk in old age. However, evidence on this topic in Asian populations remains sparse. We examined the association between weight change and all-cause mortality in a nationally-representative sample of community-dwelling older Japanese. Data came from the National Survey of the Japanese Elderly (N = 4,869, age ≥ 60 years). Participants were followed for up to 30 years. Short-term (3 years) and medium-term (6 years) weight changes were classified as “loss ≥ 5%,” “loss 2.5%–4.9%,” “stable,” “gain 2.5%–4.9%,” and “gain ≥ 5%.” Cox proportional hazards models were used to assess the relative (stable weight as reference) mortality risk associated with weight change categories. Short-term weight loss ≥ 5% was associated with higher mortality compared to the stable category, after adjusting for sociodemographic factors, health behaviors, and health conditions (hazard ratio = 1.36; 95% confidence interval = 1.22–1.51). The other weight change categories had no significant association with mortality. This was observed both among males and females. Moreover, the same pattern of results was observed when we used the medium-term weight change indicator. In conclusion, we found that both short- and medium-term weight loss greater than 5% increased the risk of dying among older Japanese; however, other types of weight change did not. This finding could inform clinical and public health approaches to body-weight management aimed to improve the health and survival of older adults, particularly in Asian populations.

